# Investigation of In Vitro Antioxidant and Antibacterial Potential of Silver Nanoparticles Obtained by Biosynthesis Using Beech Bark Extract

**DOI:** 10.3390/antiox8100459

**Published:** 2019-10-08

**Authors:** Corneliu Tanase, Lavinia Berta, Năstaca Alina Coman, Ioana Roșca, Adrian Man, Felicia Toma, Andrei Mocan, László Jakab-Farkas, Domokos Biró, Anca Mare

**Affiliations:** 1 Department of Pharmaceutical Botany, “George Emil Palade” University of Medicine, Pharmacy, Sciences and Technology of Târgu Mureș, 38 Gheorghe Marinescu Street, Târgu Mureș, 540139 Mureș, Romania; rosca_ioana01@yahoo.com; 2 Department of General and Inorganic Chemistry, “George Emil Palade” University of Medicine, Pharmacy, Sciences and Technology of Târgu Mureș, 38 Gheorghe Marinescu Street, Târgu Mureș, 540139 Mureș, Romania; lavinia.berta@umfst.ro; 3 Department of Microbiology, “George Emil Palade” University of Medicine, Pharmacy, Sciences and Technology of Târgu Mureș, 38 Gheorghe Marinescu Street, Târgu Mureș, 540139 Mureș, Romania; anca.mare@umfst.ro (A.M.); felicia.toma@umfst.ro (F.T.); adrian.man@umfst.ro (A.M.); 4 Department of Pharmaceutical Botany, ”Iuliu Hațieganu” University of Medicine and Pharmacy, 23 Gheorghe Marinescu Street, 400337 Cluj-Napoca, Romania; 5 Laboratory of Chromatography, Institute of Advanced Horticulture Research of Transylvania, University of Agricultural Sciences and Veterinary Medicine, 400372 Cluj-Napoca, Romania; 6 Faculty of Technical and Human Sciences, Sapientia Hungarian University of Transylvania, 540485 Târgu Mureș, Romania; jflaci@ms.sapientia.ro (L.J.-F.); dbiro@ms.sapientia.ro (D.B.)

**Keywords:** antioxidant, antibacterial, beech bark, silver nanoparticles, polyphenols

## Abstract

Green synthesis is one of the rapid and best ways for silver nanoparticles (AgNP) synthesis. In the present study, synthesis and bioactivity of AgNPs has been demonstrated using water beech (*Fagus sylvatica* L.) bark extract. The physical and chemical factors such as time, metal ion solution, and pH, which play a vital role in the AgNPs synthesis, were assessed. The AgNPs were characterized by ultraviolet-visible (UV-Vis) spectrometry, Fourier transform infrared spectroscopy (FT-IR), and transmission electron microscopy (TEM). Antioxidant and antimicrobial activity of the obtained AgNPs was evaluated. AgNPs were characterized by color change pattern, and the broad peak obtained at 420–475 nm with UV-Vis confirmed the synthesis of AgNPs. FT-IR results confirmed that phenols and proteins of beech bark extract are mainly responsible for capping and stabilization of synthesized AgNPs. TEM micrographs showed spherical or rarely polygonal and triangular particles with an average size of 32 nm at pH = 9, and 62 nm at pH = 4. Furthermore, synthesized AgNPs were found to exhibit antioxidant activity and have antibacterial effect against *Staphylococcus aureus,* methicillin-resistant *Staphylococcus aureus* (MRSA), *Escherichia coli*, and *Pseudomonas aeruginosa*. These results indicate that bark extract of *F. sylvatica* L. is suitable for synthesizing stable AgNPs, which act as an excellent antimicrobial agent.

## 1. Introduction

Lately, nanotechnology focuses on synthesis and characterization of metallic and non-metallic nanoparticles having different compositions, sizes, and shapes. Molecular aggregates with dimensions between 1 and 100 nm are called nanoparticles [1]. Due to their optical, magnetic, chemical, and mechanical properties, nanoparticles are used in many areas of advanced technology such as the electronic and optoelectronic industry; in the medical sector for diagnostics, antimicrobial properties, and transport of the drug to a specified location in order to fulfill its purpose; and for environmental protection and energy conversion [2]. The biosynthesis of nanomaterials becomes a large scientific area, because nanoparticles have a low obtaining cost and a wide range of uses: catalytic, biological and biomedical applications, physics, environmental remediation fields [3]. From all the types of nanoparticles, silver ones are the most commonly used. However, silver is known to be a good antimicrobial being used as an antiseptic agent [4].

The use of plant extracts in nanoparticle biosynthesis is advantageous because most plant extracts have a high content of bioactive compounds such as: flavonoids, terpenoids, tannins, and alkaloids responsible for reducing nanoparticles [5,6]. Silver nanoparticles (AgNPs) obtained by biosynthesis are characterized by different shapes and sizes, depending on the nature and concentration of the extract, pH, temperature, reaction time, and silver solution [7]. For example, flavonoids can chelate and actively reduce metal ions in nanoparticles due to the numerous hydroxyl and carbonyl groups [8]. In a study using leaf extracts from *Magnolia kobus* and *Diospyros kaki*, it has been observed that the presence of terpenoids and reducing sugars has led to the synthesis of nanoparticles, whose size and shape may vary by changing the reaction conditions [9]. Fruit extracts of *Solanum virginianum* have a high content of steroidal alkaloids: solanocarpine, carpesterol, and solanocarpidin [10]. Among the high quantity flavonoids are apigenin and quercetin which are considered as anti-capping agents and stabilizers of the formed nanoparticles [10]. In order to elucidate the mechanism of nanoparticle biosynthesis, Mittal et al. used extracts of *Syzygium cumini* fruit, observing that the presence of flavonic compounds led to the reduction of the metal ions through a redox reaction. Furthermore, the biomolecules present in the extract were responsible for AgNP aggregation and stabilization [11].

The shape and size of the biosynthesized nanoparticles depend on physical and chemical factors [12]. On the other hand, the extract used is also dependent on certain factors: the type of plant, the extraction solvent, and the extraction temperature [13,14]. In a study of turmeric extracts, Sathishkumar et al. confirmed that pH plays an important role in controlling the size and shape of the obtained nanoparticles. An alkaline plant extract has several functional groups that will facilitate the reduction process and, thus, a larger number of nanoparticles will be formed [15]. However, the optimum pH varies depending on the nature of the used ion solution.

In an experimental study using aqueous extracts of *Myrtus communis* as a reducer in nanoparticle biosynthesis, it was shown that the obtained nanoparticles have the ability to neutralize free radicals [15]. Furthermore, the ability to reduce Fe^3+^ compared to vitamin C was tested and it was observed that the reducing power increases with the increase of phenolic content [16]. However, using night jasmine extracts (*Cestrum nocturnum*), the obtained AgNPs have an antioxidant capacity almost 5% higher than that of vitamin C [17].

The increasingly common antibiotics resistance has led to the research of alternative treatment methods. Thus, in order to demonstrate the antibacterial activity, different bacteria were inoculated on culture media and it was observed that AgNPs can inhibit their growth. Inhibition was observed in comparison with the control and the strongest inhibitory activity was demonstrated on *Citrobacter* spp., *Salmonella typhi*, *Vibrio cholerae* [17]. In a recent study [18], AgNPs were synthesized from *Lantana camara*. The results showed significant antimicrobial activity against *Staphylococcus aureus, Pseudomonas aeruginosa,* and *Escherichia coli.* These results are comparable with the used standard (ciprofloxacin), but also with petroleum ether extract and essential oil from *L. camara* leaves, showing that nanoparticles have dose-dependent membrane permeation with respect to rate [18].

Taking into account the literature data, the main objectives of the current study were: (1) The biosynthesis of AgNPs using beech bark polyphenolic extract as a new bioresource; (2) the determination of the influence of the main factors on the biosynthesis process (type of reducing agent, time, and pH); (3) the characterization of the biosynthesized AgNPs by specific analyses (UV–Vis, FT-IR, and TEM); (4) the evaluation of the antioxidant and antibacterial activity of the biosynthesized AgNPs.

## 2. Materials and Methods 

### 2.1. Materials

The beech bark used for experimental research comes as waste from a wood processing company from Vatra Dornei, Romania. Before the extraction process, the bark was dried under normal temperature and aeration conditions (room temperature), and ground using a grinding mill type VEB NOSSENER MASCHINENBAU 1980. After grinding, the plant product was sieved through sieve 5.

Silver nitrate (AgNO_3_, M—169.87 g/mol, d—4.35 g/cm³), silver acetate (AgC_2_H_3_O_2_, M—166.91 g/mol, d—3.26 g/cm³), Folin–Ciocalteu reagent, potassium bromide (KBr), and methanol were purchased from Merck Company (Darmstadt, Germany), 2,2’-azino-bis (3-ethylbenzothiazoline-6-sulphonic acid)-(ABTS) was from Sigma-Aldrich (Steinheim, Germany), 2,2-diphenyl-1-picrylhydrazyl (DPPH) and 6-hydroxy-2,5,7,8-tetramethylchroman-2-carboxylic acid (Trolox) were obtained from Alfa-Aesar (Karlsruhe, Germany).

The following bacterial reference strains, stored at −70 °C in the Microbiology Laboratory from UMFST Târgu Mureș, were used: *S. aureus* ATCC (American Type Culture Collection) 25923, methicillin-resistant *S. aureus* (MRSA) ATCC 43300, *E. coli* ATCC 25922, *Klebsiella pneumoniae* ATCC 700603, *P. aeruginosa* ATCC 27853.

### 2.2. Extraction Method

The ultrasound-assisted extraction was made according to the method described previously [19] with some modifications. Briefly, 10 g of beech bark was placed in an Erlenmeyer flask. Then, 100 mL of distilled water was added. The Erlenmeyer flask was then inserted into the ultrasonic water bath (Professional Ultrasonic Cleaner MRC: AC 150 H, 150 W, 40 KHz, heating power 300 W) preheated to 70 °C, for an extraction time of 30 min. At the end of the extraction, the solution obtained was filtered through the Buchner cone, transferring the filtrate to a 100 mL volumetric flask and filled up to the mark with distilled water. In order to obtain a clear solution and to avoid the interference of any bark residues in the biosynthesis process, the extract was centrifuged in centrifuge tubes, for 5 min at 5000 rpm.

### 2.3. Characterization of Extract

The determination of the polyphenol content was performed spectrophotometrically, using the Folin–Ciocalteu method [20]. The total polyphenol content (TPC), expressed in mg gallic acid (GAE)/g plant material, was calculated based on the absorbance read with the spectrophotometer at 765 nm, taking into account the calibration curve (concentration range between 0 and 200 mg/L) of the standard gallic acid solution.

### 2.4. Synthesis of Silver Nanoparticles

For the biosynthesis, 100 mL of silver nitrate/acetate solution (1 mM) was prepared in advance. For adjusting the pH, a 50 mL HNO_3_/ NaOH solution (1 mM) was prepared. Biosynthesis consists of mixing 10 mL of beech bark extract with 90 mL of silver nitrate/acetate in an Erlenmeyer glass. From the obtained solution, the pH was measured, and the values were in the range of 5.56–5.63, depending on the experimental version. Using the pH meter with an agitator at 800 rpm the solutions were brought to pH = 4 using about 4 mL HNO_3_, and to pH = 9 using about 5 mL NaOH, depending on the initial pH of the solution. Biosynthesis took place in the ultrasonic bath at 60 °C, for 3 h until the color transformation indicated finalization of nanoparticles biosynthesis. At the time of introducing the samples into the ultrasonic water bath, samples were taken for each experimental version in order to record the spectrum of the initial solution. Tested solutions (TS) were: TS1—beech bark extract, pH = 4, silver nitrate; TS2—beech bark extract, pH = 9, silver nitrate; TS3—beech bark extract, pH = 4, silver acetate; TS4—beech bark extract, pH = 9, silver acetate.

### 2.5. Characterization of Silver Nanoparticles

#### 2.5.1. UV-Vis Analysis 

The samples of synthesized nanoparticles were measured at different time intervals (T0 = 0, T1 = 1 h, T2 = 1 h 30’, T3 = 2 h, T4 = 3 h, T5 = 24 h) based on previous results (in publication process). The results were recorded with the Analytik Jena Specord 210 Plus190 (UV/Vis)-1100 nm Spectrophotometer using synthetic quartz spectrophotometer cells (190–2500 nm, path length 10 mm, volume 1.75 mL). The measurements were performed in the following parameters: 1 nm resolution, the wavelength range between 350 and 700 nm. 

#### 2.5.2. FT-IR Analysis

For the FT-IR analysis, the AgNP dry matter was used. FT-IR analysis involves mixing the obtained powder with KBr in the agate mortar in a ratio of 1:100. Using the FT-IR Thermo Nicolet 380 Spectrophotometer (Thermo Scientific, Waltham, USA), the measuring range 4000–400 cm^−1^, spectra were recorded for each TS.

#### 2.5.3. TEM Analysis

The nanoparticles in the solution were characterized morphologically and dimensionally using conventional electron transmission microscopy, using a JEOL 100 U TEM microscope (Japan Electron Optics Laboratory, Tokyo, Japan) at 100 kV. The nitrocellulose substrates coated with amorphous carbon layers, prepared on a 300 mesh copper microgrid, served as the substrate used for nanoparticle investigation. The thickness of the thermally evaporated carbon layer was about 4 nm. The SERS (Surface-Enhanced Raman Scattering) spectra were recorded in solution with a portable spectrometer (Raman Systems R3000 CN, Edinburgh, United Kingdom) equipped with a 785 nm diode coupled to a 100 km optical fiber. The laser power was 200 mV and the integration time was 30 s.

The obtained images were processed with the ImageJ software to determine the AgNP size and the histograms were made for each experimental version with Excel.

### 2.6. In Vitro Antioxidant Activities

#### 2.6.1. Free Radical-Scavenging Activity Using 2,2-Diphenyl-1-picrylhydrazyl (DPPH)

The antioxidant activity was monitored according to the DPPH method described by Martins et al. [21] with modifications. This method was performed by using a SPECTRO star Nano microplate reader (BMG Labtech, Offenburg, Germany). The reaction mixture in each of the 96-wells consisted of 30 µL of sample solution (in an appropriated dilution, according to the reference range of the calibration curve) and a 0.004% methanolic solution of DPPH. The mixture was further incubated for 30 min in the dark, and the reduction of the DPPH radical was determined by measuring the absorption of the sample at 515 nm. Trolox was used as a standard reference and the results were expressed as Trolox equivalents (TE) per g of dry weight (mg TE/g dw TS).

#### 2.6.2. Trolox Equivalents Antioxidant Capacity (TEAC) Assay 

The radical scavenging activity of the tested solutions against the stable synthetic 2,2’-azino-bis 3-ethylbenzothiazoline-6-sulphonic acid (ABTS) radical cation was measured using the method previously described by Mocan et al. [22]. Briefly, 20 µL of sample is mixed with 200 µL of radical solution and incubated for 6 min. The absorbance of the final solution is measured at 760 nm after the incubation period. A Trolox calibration curve was plotted as a function of the percentage of ABTS radical scavenging activity. The final results were expressed as milligrams of TE per gram of dry weight tested solutions (mg TE/g dw TS).

### 2.7. Antibacterial Activity

#### 2.7.1. Minimal Inhibitory Concentration (MIC) of Silver Nanoparticles

In order to determine the MICs of the obtained AgNPs against tested bacterial strains, we used the microdilution method, as previously described [20]. To obtain binary dilutions from the TS, 200 µL TS was added in the first column of the microplate. One-hundred microliters of sterile water were added in all the other wells of the microplate. One-hundred microliters from the first column were transferred with a multichannel pipette into the second column of the microplate. These steps were repeated until the last column, from which, the last 100 µL was discarded. Ten microliters of 0.5 McFarland bacterial suspension were mixed with 9990 µL of Mueller-Hinton broth medium 2X and 100 µL from this bacterial inoculum were dispensed in each well of the microplate. Additionally, wells with culture medium only, culture medium with TS (negative controls), and culture medium with bacterial inoculum (growth control) were prepared. The microplates were incubated at 37 °C for 24 h. MIC was considered in the last well in which no bacterial growth was noted. For TS with a high degree of turbidity, resazurin was used as an indicator of bacterial growth [23]. After the plates were incubated for 24 h at 37 °C, 3 µL 0.015% resazurin was added to each well, and the plates were further incubated for 2–4 h. A color change of resazurin, from purple to pink, indicated a bacterial growth. The last well in which the resazurin color did not change was considered MIC. 

#### 2.7.2. Minimal Bactericidal Concentration (MBC) of Silver Nanoparticles

From each well in which no bacterial growth was observed in the MIC method, 1 µL of suspension was spot-inoculated on blood agar with a calibrated bacteriological inoculation loop. MBC was considered the first TS concentration where no bacterial growth was observed on blood agar.

#### 2.7.3. AgNP Effect on Bacterial Growth Rate (GR)

The following bacterial strains were used: *S. aureus* ATCC 25923 (SA); *E. coli* ATCC 25922; *P. aeruginosa* ATCC 27853. To determine the growth rate, the solution with the highest bactericidal activity was chosen (TS4). For the determination of GR, the steps described in a previous work were performed [20]. The content of the microplate well in which MIC was noted was reproduced in a 2 mL Eppendorf tube and it was incubated at 37 °C. At the initial moment and after 3 and 6 h of incubation, 50 µL of bacterial suspension were removed, serial diluted, and from these dilutions, 50 µL were evenly seeded on the surface of Mueller-Hinton agar with a bacteriological inoculation loop. The plates were incubated for 18–24 h and the colonies were automatically counted using “Flash & Go Automatic Colony Counter” instrument (IUL Instruments S.A., Bacelona, Spain) from the plate with the most countable colonies. As control for the bacterial growth, the same protocol was used, in the absence of TS. Mathematical adjustments were performed to compensate the dilution from where the colonies were counted and the inoculation volume. 

### 2.8. Statistical Analysis

All analytical determinations were performed in triplicate, and the results are expressed as the mean ± standard deviation. The statistical significance was assessed by GraphPad InStat 3 software (GraphPad Software, San Diego, Canada), at a significance threshold value of *p* < 0.05.

## 3. Results

### 3.1. Characterization of Aqueous extracts 

The aqueous extract was characterized in terms of total phenolic content (TPC), which is considered responsible for silver ion reduction and AgNP synthesis. Thus, the total phenolic content was found to be 20.59 mg GAE/g plant material. 

### 3.2. Characterization of AgNPs

**Figure 1:**
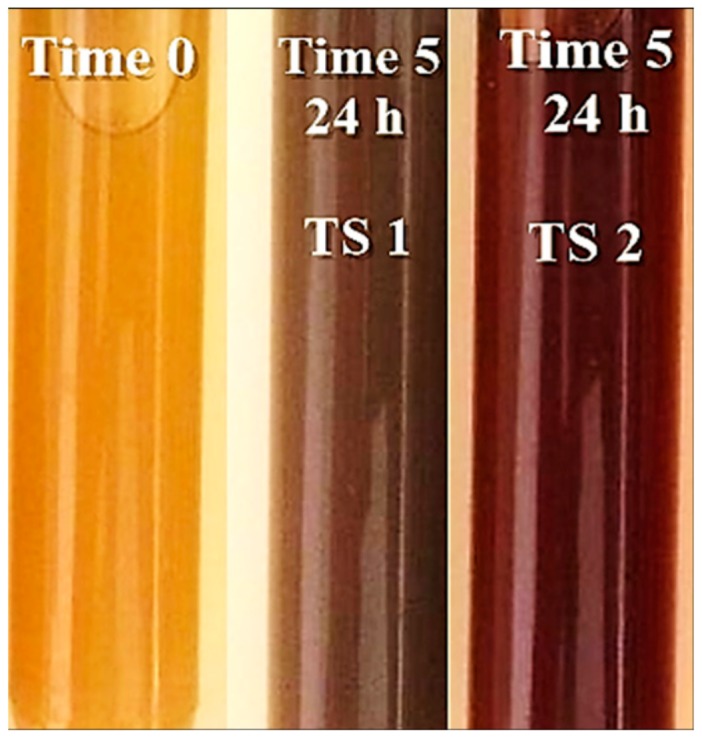
Color transformation of beech bark extract solution with AgNO_3_ at pH = 4 (test solution 1 (TS1)) and pH = 9 (TS2).

The first sign of nanoparticle formation is the color transformation of the solution. The influence of the extract and of the used pH has an important role and it is observed that the solutions change their color becoming brownish-red at pH = 9 and brown at pH = 4 (Figure 1). The appearance of the opaque aspect is achieved as the biosynthesis time passes.

#### 3.2.1. UV-Visible Spectral Analysis of AgNPs

**Figure 2:**
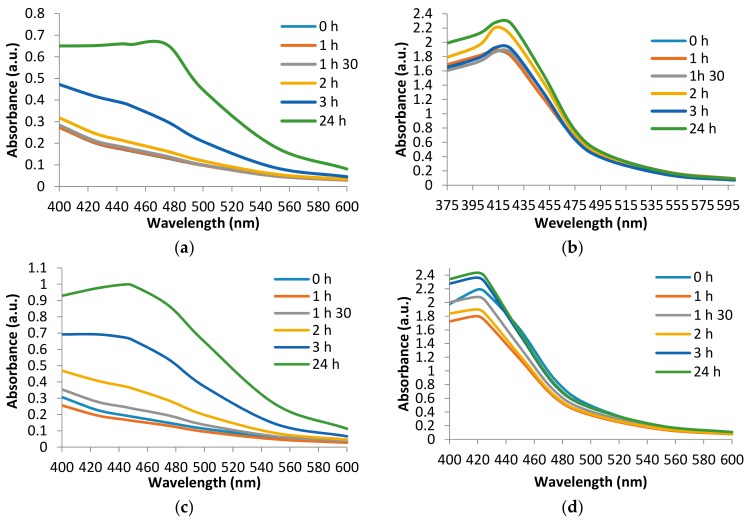
UV–visible absorption spectra of synthesized silver nanoparticles using tested solutions: (**a**) TS1—beech bark extract, pH = 4, AgNO_3_; (**b**) TS2—beech bark extract, pH = 9, AgNO_3_; (**c**) TS3—beech bark extract, pH = 4, AgC_2_H_3_O_2_; (**d**) TS4—beech bark extract, pH = 9, AgC_2_H_3_O_2_.

The optical and electronic properties of the obtained silver nanoparticles are observed by UV-Vis spectrophotometric analysis. Many factors that influence the absorption spectrum shift have been analyzed, observing that the wavelength at which the maximum absorbance is recorded is dependent on the time, pH, and silver ion solution. The biosynthesis time, visually determined, is 3 h for each experimental version, but the time of appearance of the nanoparticles is different and can be observed by UV-Vis spectra analysis (Figure 2). Use of beech bark extract (TS1) after 3 h of maintenance in the ultrasonic bath leads to the formation of AgNPs. The maximum absorbance was recorded at the wavelength of 475 nm (Figure 2a). The recording of the UV-Vis spectrum for TS2 shows a maximum absorbance corresponding to the wavelength 425 nm from time 0 (Figure 2b). For TS3, it can be observed that the time of appearance of AgNPs is 3 h and the maximum absorbance corresponds to the wavelength of 450 nm (Figure 2c). At basic pH (TS4), under the same experimental conditions it is observed (Figure 2d) that the appearance of the maximum absorbance corresponds to the wavelength 420 nm.

UV-Vis spectra recordings show that the wavelengths recorded for the TS are mainly pH dependent. Thus, at pH = 4, maximum absorbances are recorded at wavelengths in the range of 450–475 nm. When using the basic pH, the UV-Vis spectra shows a shift of the maximum absorbances at a wavelength range between 411 and 431 nm. These results are supported by the literature data in which the use of acidic pH records lower maximum absorbances [15]. The value of the acidic pH leads to the agglomeration of the nanoparticles. Thus, on the UV-Vis spectrum, there is a lower absorbance peak compared to the use of a basic pH which favors obtaining a larger number of nanoparticles and smaller dimensions. Thus, at basic pH, the reaction rate increases followed by crystallization into smaller particles, which involves the nucleation process and the growth of small nanoparticles [15,24]. In a study using extracts from oak bark, rich in polyphenolic compounds and tannins, it was shown that the synthesis rate of AgNPs increases with increasing pH. The pH with value 9 has the highest absorbance; above this value the synthesis rate decreases [25]. Analyzing the experimental versions comparatively, depending on the used silver solution, no significant differences were found.

#### 3.2.2. FT-IR Analysis of Biosynthesized AgNP

**Figure 3:**
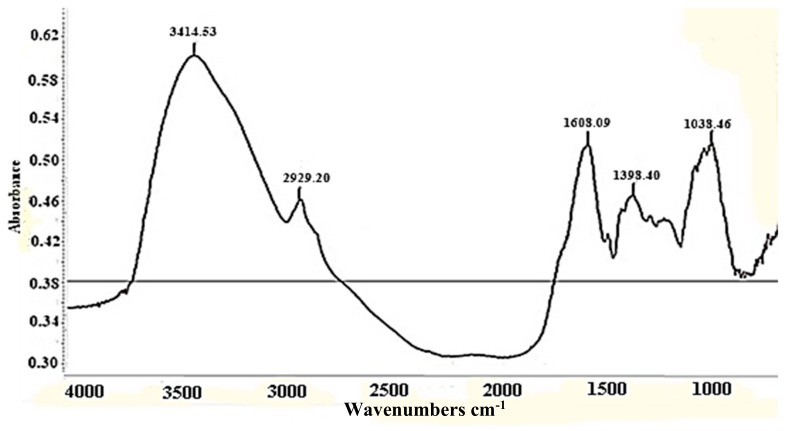
Fourier transform infrared spectra of aqueous bark extract.

FT-IR analyses were conducted to identify the biomolecules that might be responsible for reducing the Ag+ ions in the beech bark extract, and also those that may be involved in the stabilization of silver nanoparticle synthesis. Figure 3 shows the spectrum of aqueous beech bark extract, where the bands corresponding to the –O–H bonds at 3414 and 1608 cm^−1^ specific to the carbonyl group can be observed. 

**Figure 4:**
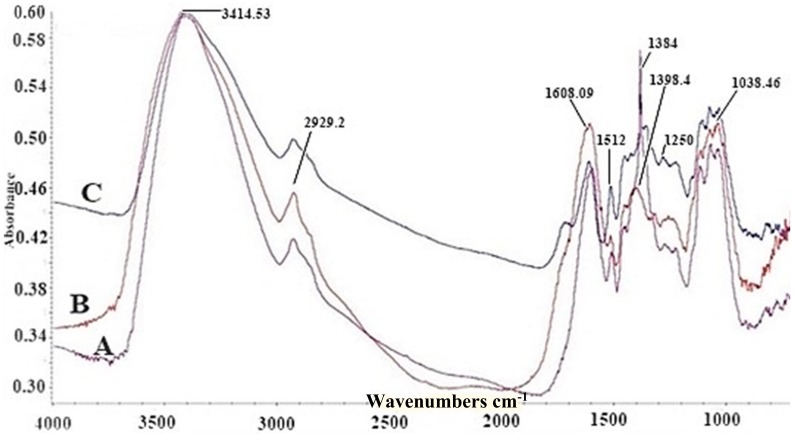
Fourier transform infrared spectra of gold nanoparticles (AgNPs): A—TS2 (AgNPs obtained with AgNO_3_ at pH = 9), B—aqueous bark extract, C—TS1 (AgNPs obtained with AgNO_3_ at pH = 4).

The recording of the FT-IR spectra in the case of AgNPs obtained by reducing AgNO_3_ (TS1–TS2) with the polyphenolic compounds in the aqueous beech extract shows the appearance of a band at the value of 1512 cm^−1^ and intensifies the band corresponding to the wavenumber 1384 cm^−1^ (Figure 4). These show that certain compounds in the aqueous extract modify their structure, thus, generating AgNPs. For TS3–TS4 solutions, there were no different results compared to TS1–TS2.

#### 3.2.3. The Analysis by Transmission Electron Microscopy (TEM) of Biosynthesized AgNP

Transmission electron microscopy was used in order to characterize the obtained nanoparticles in terms of their morphology and size. Thus, the TEM photomicrographs obtained were analyzed using ImageJ program, obtaining information about the surface and diameter of the biosynthesized nanoparticles.

**Figure 5:**
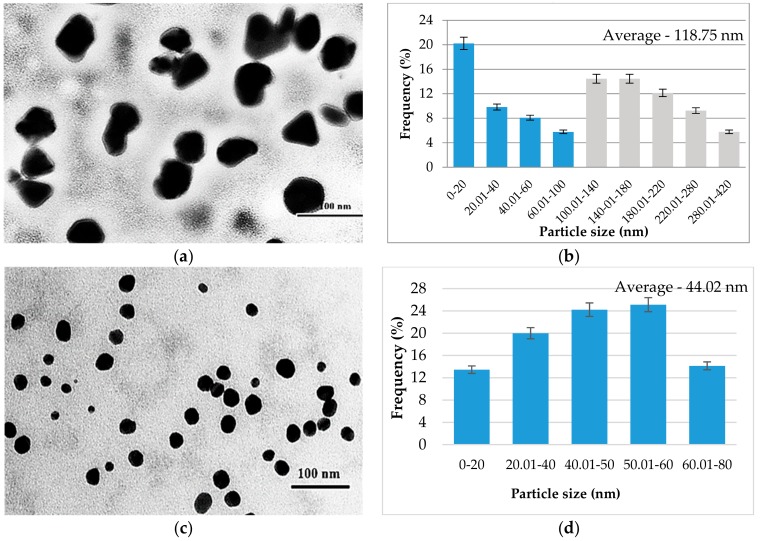
Graphical representation of AgNPs synthesized in the presence of the extract obtained from the beech bark and AgC_2_H_3_O_2_: (**a**) TS3— TEM photomicrograph; (**b**) histogram of the distribution of AgNP size distribution in TS3; (**c**) TS4—TEM image; (**d**) histogram of the distribution of AgNP size distribution in TS4. (TS3—beech bark extract, pH = 4, AgC_2_H_3_O_2_; TS4—beech bark extract, pH = 9, AgC_2_H_3_O_2_).

In Figure 5, it is observed that the AgNPs synthesized are nanometric and uniformly distributed. Regarding the morphology of the nanoparticles, it can be observed that it varies, encountering polygonal, spherical, and even triangular shapes at TS1 (Figure 5a). In contrast, at basic pH (TS2) only spherical shapes are observed (Figure 5c). The particles synthesized in TS1 have sizes between 10 and 420 nm (Figure 5b), with an average of 118.75 nm, and about 44% of these have a diameter below 100 nm. Regarding the size of the nanoparticles obtained at pH 9 (TS2) they fall in the range of 2–80 nm, with an average of 44.02 nm and 100% of the measured nanoparticles have dimensions smaller than 100 nm (Figure 5d). 

**Figure 6:**
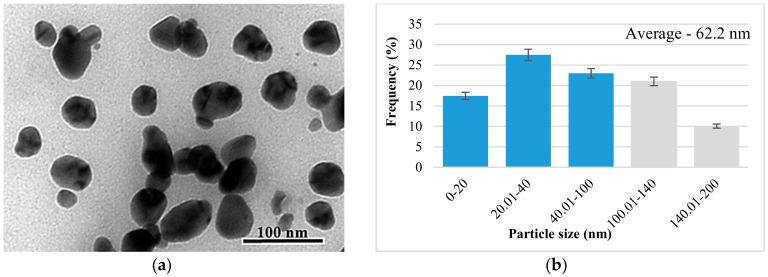

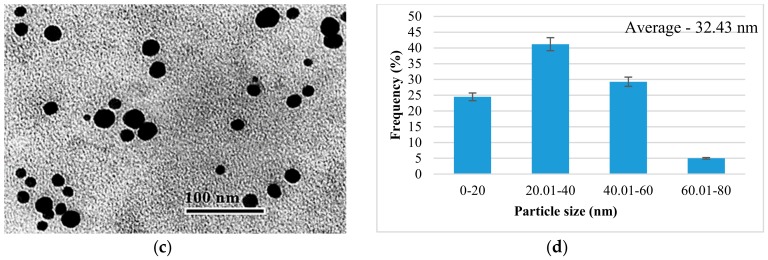
Graphical representation of AgNP synthesized in the presence of the extract obtained from the beech bark and AgNO_3_: (**a**) TS1— TEM photomicrograph; (**b**) histogram of the distribution of the AgNP size distribution in the TS1; (**c**) TS2—TEM image; (**d**) histogram of the distribution of AgNP size distribution in TS2 (TS1—beech bark extract, pH = 4, AgNO_3_; TS2—beech bark extract, pH = 9, AgNO_3_).

Analyzing the synthesized AgNPs in the presence of the extract obtained from the beech bark and AgNO_3_ (Figure 6), the results are similar to those presented previously. The morphology of the obtained AgNPs at pH = 4 (TS3) is varied, in this case observing polygonal, triangular, and spherical shapes (Figure 6a). In contrast, at basic pH (TS4) it can be observed that the AgNP morphology is constant, with only the spherical shape being met (Figure 6c). AgNPs obtained in the basic medium have dimensions in the range of 2–80 nm, with an average of 32.43 and 100% being smaller than 100 nm in diameter (Figure 6d).

The results obtained with the help of TEM confirm the results presented above, regarding the basic pH which favors the formation of a larger number of smaller nanoparticles. Thus, comparing the tested solution, it is found that at pH = 9 all AgNPs have dimensions below 100 nm, and at pH = 4, about half of AgNPs have dimensions below 100 nm. These findings, related to shape and size, are also confirmed by other researchers who analyzed synthesized nanoparticles in the presence of extracts obtained from the bark of woody plants [26,27]. The particles synthesized in TS3 have sizes between 10 and 200 nm (Figure 6b), with an average of 62.2 nm.

Thus, from literature data, AgNPs with a larger surface area, provide better contact with microorganisms [28]. These particles are able to penetrate into the cell membrane or attach to the bacterial surface based on their size. In addition, they have been reported to be highly toxic to bacterial strains and their antibacterial efficacy is increased by decreasing the particle size [29].

## 4. Antioxidant Activity of AgNPs

The antioxidant activity of synthesized AgNPs was evaluated by DPPH and TEAC radical scavenging assays. The beech bark AgNPs exhibited free-radical-scavenging activity using both assays as shown in Table 1. The activity against DPPH and ABTS confirmed the antioxidant potential of the beech bark AgNPs. Significantly higher TEAC values were noted for TS1–TS2 as compared to TS3–TS4. Many AgNPs solutions have been evaluated for their antioxidant capacities, commonly associated with the content of phenolic compounds from plant extracts [17,30,31]. Beech bark contains a range of biologically active components, such as tannins and polyphenols, indicating that the antioxidant activity of the AgNPs has been enhanced by these bio-components.

## 5. Antimicrobial Activity of AgNPs

To evaluate the antibacterial activity of the AgNPs against gram-negative bacteria (*E. coli* ATCC 25922, *K. pneumoniae* ATCC 700603, *P. aeruginosa* ATCC 27853) and gram-positive ones (*S. aureus* ATCC 25923 and MRSA ATCC 43300), MIC and MBC values were obtained. Afterwards, we studied how the TS influenced the bacterial growth rate (at the initial moment and after 3 and 6 h of incubation).

For therapeutic consideration, only antimicrobial activity obtained below 1 mM concentration was considered. The MIC and MBC of nanoparticles against pathogenic bacterial strains are as mentioned in Table 2. Thus, it is found that almost all tested solutions exhibit inhibitory activity on the growth of tested bacteria (except TS1, where the MIC was determined only for MRSA, at the maximum analyzed concentration). It can be seen that AgNPs obtained at pH = 9 (TS2, TS4) favor the inhibitory activity for the tested bacterial strains. They have a lower MIC compared to those obtained at pH = 4 (TS1, TS3). TS2 (for *E. coli*, *K. pneumoniae*, and *P. aeruginosa*) and TS4 (for *S. aureus*, *E. coli*, *K. pneumoniae*, and *P. aeruginosa*) have lower MIC and MBC compared to those obtained for the control beech bark extract. For the most part, the MBC was registered for the TS where the MIC was determined, the results being presented in Table 2. The lowest MBC was recorded for TS4 (MRSA, *E. coli*, and *P. aeruginosa*) and for TS2 (*E. coli*).

**Figure 7:**
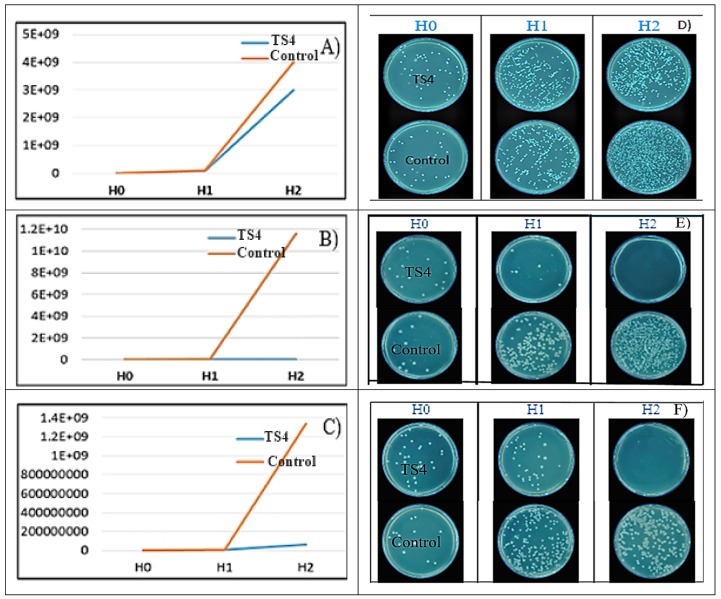
Graphical and visual representation of the growth rate for *S. aureus* (**A**,**D**), *E. coli* (**B**,**E**), *P. aeruginosa* (**C**,**F**) in the presence of TS4 (beech extract + AgC_2_H_3_O_2_ pH = 9) and in the absence of TS4 (Control) at: initial time—H0, 3 h—H1, and 6 h—H2.

As can be seen from Figure 7, TS4 had a bactericidal effect on *E. coli* and inhibited the growth of *S. aureus* and *P. aeruginosa* after 6 h of incubation. 

The antibacterial mechanism might beexplained by the interaction between silver nanoparticles and bacterial DNA. AgNPs can cause DNA damage, and ultimately bacterial cell death. This effect has been demonstrated on *S. aureus* and *E. coli* strains [32].

However, the antibacterial mechanism is different, depending on the type of bacteria to be inhibited. For example, silver nanoparticles have an inhibitory effect on *Helicobacter pylori*, stating two theories of action: small amounts of AgNPs enter the bacterium and inhibit the respiratory chain, while another theory considers that AgNPs inhibit urease from *H. pylori* [33]. Evidence of bacterial growth inhibition have also been demonstrated for strains of *S. aureus*, *Streptococcus pneumoniae*, *Proteus mirabilis*, and *E. coli* [34].

## 6. Conclusions

Following the performed analyses, it was found that the biosynthesis of silver nanoparticles is time and pH dependent. UV-Vis analysis shows that basic pH accelerates biosynthesis independently of the ion salt used. This is also supported by TEM analysis in which the appearance in large quantities and smaller size of AgNPs was demonstrated in all the versions where the basic pH was used.

FTIR analysis demonstrated that certain functional groups of the compounds present in the aqueous extracts contributed to the formation of AgNPs, a fact supported by the intense bands around 1384 cm^−1^. 

AgNPs tested solutions showed antioxidant activity as well as inhibitory and bactericidal activity. The lowest MBC was obtained for TS4 (0.19 mg/mL) against *E. coli* ATCC 25922. The small nanoparticles at pH = 9 have a lower MIC/MBC compared to those synthesized at pH = 4. This result could be explained by the fact that alkaline pH favors the penetration of AgNPs into the bacterial cell, having an inhibitory or bactericidal effect.

By determining the growth rate, it was observed that for *E. coli*, the tested AgNPs had a bactericidal effect; while for *S. aureus* and *P. aeruginosa* the growth was inhibited.

These results indicate that bark extract of *Fagus sylvatica* L. is suitable for synthesizing stable AgNPs which act as an antimicrobial agent.

## Figures and Tables

**Table 1:** Antioxidant activity of AgNPs.

Sample	Beech Bark AgNP Characteristics	DPPH mg TE/g of Sample	TEAC mg TE/g of Sample
**TS1**	pH = 4, AgNO_3_	11.68 ± 0.34	35.05 ± 0.69
**TS2**	pH = 9, AgNO_3_	10.64 ± 0.35	37.23 ± 0.35
**TS3**	pH = 4, AgC_2_H_3_O_2_	10.52 ± 0.21	28.61 ± 0.21
**TS4**	pH = 9, AgC_2_H_3_O_2_	8.52 ± 0.15	20.39 ± 0.30

TE—Trolox equivalents; TS1—beech bark extract, pH = 4, AgNO_3_; TS2—beech bark extract, pH = 9, AgNO_3_; TS3—beech bark extract, pH = 4, AgC_2_H_3_O_2_; TS4—beech bark extract, pH = 9, AgC_2_H_3_O_2_. ± Standard deviation.

**Table 2:** Antimicrobial activity of AgNPs against pathogenic bacteria.

Pathogenic Bacteria	ATCC No.	AgNP Tested Solution	MIC	MBC
			mg/mL	mg/mL
*Staphylococcus aureus*	25923	TS1	>1.41	>1.41
		TS2	0.27	3.28
		TS3	1.21	>1.45
		TS4	0.09	2.37
		BBE	>2.5	>2.5
		AgNO_3_	0.02	>0.15
		AgC_2_H_3_O_2_	0.03	>0.15
MRSA	43300	TS1	1.41	1.41
		TS2	0.34	0.82
		TS3	0.42	1.45
		TS4	0.24	0.79
		BBE	>2.5	>2.5
		AgNO_3_	0.02	0.05
		AgC_2_H_3_O_2_	0.02	0.05
*Escherichia coli*	25922	TS1	>1.41	>1.41
		TS2	0.54	0.68
		TS3	1.45	1.45
		TS4	0.19	0,19
		BBE	>2.5	>2.5
		AgNO_3_	0.02	0.02
		AgC_2_H_3_O_2_	0.02	0.02
*Klebsiella pneumoniae*	700603	TS1	>1.41	>1.41
		TS2	2.74	3.28
		TS3	1.45	1.45
		TS4	0.99	1.38
		BBE	>2.5	>2.5
		AgNO_3_	>0.15	>0.15
		AgC_2_H_3_O_2_	>0.15	>0.15
*Pseudomonas aeruginosa*	27853	TS1	>1.41	>1.41
		TS2	0.41	0.82
		TS3	1.45	>1.45
		TS4	0.15	0.25
		BBE	>2.5	>2.5
		AgNO_3_	0.02	0.02
		AgC_2_H_3_O_2_	0.02	0.02

ATCC—American Type Culture Collection; MIC—minimal inhibitory concentration; MBC—minimum bactericidal concentration; MRSA—methicillin-resistant *S. aureus*, BBE—beech bark extract; AgNO_3_—silver nitrate; AgC_2_H_3_O_2_—silver acetate; TS1—beech bark extract, pH = 4, AgNO_3_; TS2—beech bark extract, pH = 9, AgNO_3_; TS3—beech bark extract, pH = 4, AgC_2_H_3_O_2_; TS4—beech bark extract, pH = 9, AgC_2_H_3_O_2._
